# Improving Survival in Patients with Decompensated Cirrhosis

**DOI:** 10.4061/2011/565108

**Published:** 2011-12-07

**Authors:** Deepak Amarapurkar, Rajiv Jalan, Richard Guan, Paul Kwo

**Affiliations:** ^1^Department of Gastroenterology, Bombay Hospital and Medical Research Centre, Mumbai 400025, India; ^2^The London Clinic Liver Centre, 116 Harley Street, London W1G 7JL, UK; ^3^Mount Elizabeth Medical Centre, Mount Elizabeth No. 17-02, Singapore 228510; ^4^Liver Transplantation, Division of Gastroenterology and Hepatology, Indiana University, School of Medicine, Indianapolis, IN 46202-5121, USA

About 60% of patients with decompensated disease have oesophageal varices. One third of these patients will experience variceal bleed. Each bleeding episode compromises the decompensated state and is associated with a 20% to 30% mortality. Bleeding from ectopic varices is rare but is generally massive and life threatening. The first two articles, “*Clinicopathological features and treatment of ectopic varices with portal hypertension*” and “*Application of endoscopy in improving survival of cirrhotic patients with acute variceal hemorrhage*” discuss the management of variceal bleeding in cirrhotic patients. “*Improved survival with the patients with variceal bleed*” is a review article on how new treatment modalities have improved the outlook of patients with bleeding oesophageal varices. 

The hepatorenal syndrome (HRS) signifies advanced liver failure and is a bad prognostic factor in patients with decompensated cirrhosis. Management of this condition is discussed in “*Management of renal failure and ascites in patients with cirrhosis*”. One of the features of decompensated liver disease is the occurrence of recurrent or resistant ascites. Transjugular intrahepatic portosystemic shunt (TIPS) is an effective therapy for refractory ascites and HRS at the expense of hepatic encephalopathy and may offer an effective bridge to liver transplantation, by improving short and medium term survivals, as discussed in “*Role of TIPS in improving survival of patients with decompensated liver disease*”. 

Bacterial infection is responsible for up to a quarter of the deaths of patients with decompensated liver disease. “*Prevention and management of bacterial infections in cirrhosis*” discuss the high index of suspicion that is needed to prevent bacterial infections in patients with decompensated cirrhosis. These patients are immunologically compromised, and prophylactic antibiotics can prevent fatal septicemia and HRS in those with gastrointestinal bleeding. Current thoughts on how to deal with the neuropsychiatric complication of cirrhosis are discussed in “*Management of hepatic encephalopathy*”. Cardiomyopathy, hepatopulmonary syndrome, portopulmonary hypertension and right-sided hydrothorax complications that are often overlooked in patients with decompensated liver disease are discussed in “*Management of cardiopulmonary complications of cirrhosis*”. Decompensated liver cirrhosis has been traditionally considered as a prototype of hemorrhagic coagulopathy, and routinely performed coagulation profile is abnormal in the majority of these patients. In “*Management of coagulopathy in patients with decompensated liver disease*”, the authors discussed recent thoughts on coagulation in end-stage liver disease. The related article entitled “*Determination of ADAMTS13 and its clinical significance for ADAMTS13 supplementation therapy to improve the survival of patients with decompensated liver cirrhosis*” reviews the role of the deficiency of the metalloproteinase ADAMTS13 in end-stage liver cirrhosis in inducing platelet clumping or thrombi and how the resulting sinusoidal microcirculatory disturbances causes further liver damage and is closely related to further deterioration of liver function, hepatic encephalopathy, hepatorenal syndrome, and intractable ascites in advanced liver cirrhosis. Fresh frozen plasma (FFP) is a source of ADAMTS13. 

Liver cirrhosis is the common end stage of persistent liver injury. In the Asia Pacific region, these injuries commonly result from chronic hepatitis B and C infections as well as alcohol. The following two articles, “*Treatment of hepatitis B in decompensated liver cirrhosis *and *treatment of decompensated alcoholic liver disease*” address the management of hepatitis B and alcoholic liver disease in end stage liver disease. Pharmacotherapy in patients with decompensated liver disease is not without complications and side effects and might compromise the decompensated state. The article entitled “*Prescribing medications in patients with decompensated liver cirrhosis*” addresses the above conundrum. A common complication of liver cirrhosis is liver cancer, and treatment of this condition is challenging in patients with liver decompensation to say the least. The paper entitled “*Screening for hepatocellular carcinoma*” discusses early detection of liver cancer in these patients so that appropriate management can be arranged. Finally, liver transplantation for end-stage liver failure is discussed in “*Indications and contraindications for liver transplantation*”.

**Deepak Amarapurkar** **Deepak Amarapurkar** **Rajiv Jalan** **Rajiv Jalan** **Richard Guan** **Richard Guan** **Paul Kwo** **Paul Kwo**

